# Opportunities and Barriers to HPV Vaccination Among Men Who Have Sex with Men and Related Sexual and Gender Minority Populations: A Systematic Review and Exploratory Clustering Analysis Using a Socio-Ecological Framework

**DOI:** 10.3390/vaccines14070632

**Published:** 2026-07-20

**Authors:** Jiayu Cai, Zhuohang Liu, You Zuo, Yiu Wing KAM

**Affiliations:** Division of Natural and Applied Sciences, Duke Kunshan University, Kunshan 215316, China; jiayu.cai@dukekunshan.edu.cn (J.C.); zhuohang.liu@dukekunshan.edu.cn (Z.L.); you.zuo@dukekunshan.edu.cn (Y.Z.)

**Keywords:** human papillomavirus, HPV vaccination, men who have sex with men, socio-ecological model, vaccine uptake

## Abstract

**Background**: Men who have sex with men (MSM) experience a disproportionate burden of human papillomavirus (HPV)-related disease, yet vaccination uptake remains uneven. With MSM as the key population of interest, this review synthesized evidence on uptake, willingness, multilevel barriers and opportunities, and vaccine-oriented outcomes among MSM and related sexual and gender minority populations when MSM-relevant findings could be extracted. **Methods**: Six databases were searched for original English- or Chinese-language studies published from 1 January 2010 to 31 December 2025. Findings were synthesized narratively. Barriers and opportunities were mapped using a five-level socio-ecological model; study-level willingness–uptake patterns were explored using K-means clustering as an exploratory analysis among the nine studies reporting both outcomes; and genotype-specific and immunogenicity findings were summarized separately. **Results**: Fifty-three studies involving 169,241 participants were included; 87.2% of participants were MSM, and 79% of studies were cross-sectional. Barriers and opportunities occurred across individual, provider-related interpersonal, organizational/institutional, community, and policy/societal levels. Recurrent paired mechanisms involved knowledge and trust, provider recommendation and communication, service accessibility, community support, and eligibility and affordability. Nine studies contributed data to the exploratory clustering analysis; seven fell into low-uptake clusters, including three with high willingness but low uptake, which may indicate a possible study-level implementation gap. Two studies reporting genotype prevalence and antibody responses provided limited biological context supporting vaccination before exposure and suggesting potential benefit from catch-up vaccination. **Discussion**: Among MSM and related sexual and gender minority populations, HPV vaccination is shaped by interacting individual and structural conditions, with MSM remaining the principal focus of the evidence base. Improving uptake is likely to require complementary strategies: universal, gender-neutral routine vaccination in adolescence before their sexual debut, and targeted catch-up for MSM who missed routine vaccination, supported by trusted provider endorsement, convenient service delivery, community engagement, and inclusive, affordable policy. The thematic counts were study-level and unweighted, and the nine-study clustering analysis was exploratory rather than definitive.

## 1. Introduction

Human papillomavirus (HPV) comprises a diverse group of double-stranded DNA viruses that infect epithelial tissues of the skin and mucosa [1]. More than 200 HPV genotypes have been identified, including over 40 types that infect the anogenital and oropharyngeal regions [2]. Low-risk types, such as HPV 6 and 11, cause genital warts, whereas high-risk types, including HPV 16 and 18, are linked to HPV-related cancers.

Persistent infection with high-risk HPV types is a major cause of anogenital and oropharyngeal cancers, and cervical cancer remains the most widely recognized HPV-related malignancy [3]. However, HPV also causes a substantial burden of disease among men, including genital warts and cancers of the anus, penis, and oropharynx [4].

Within male populations, men who have sex with men (MSM) are a key group for HPV prevention because they experience higher HPV infection and HPV-related disease burden than the general male population [5]. Anal cancer is strongly associated with oncogenic HPV types, and the reported incidence among MSM is substantially higher than that in the general male population [6]. This elevated risk may be reinforced by HIV co-infection, network-level transmission, and limited indirect protection from female-only vaccination programmes.

Despite this risk, HPV vaccination coverage among males remains suboptimal in many settings. The historical framing of HPV as a women’s health issue has contributed to lower awareness and uptake among male populations [7]. Recent work has emphasized the need to improve vaccination implementation, including simplified schedules and broader public health promotion, to increase access and coverage [8]. For MSM, the challenge is not only whether individuals are willing to be vaccinated, but also whether information, provider recommendation, service access, community support, and policy conditions allow willingness to translate into uptake.

Existing evidence on HPV vaccination among MSM-relevant sexual and gender minority populations is geographically and methodologically diverse. Studies have examined uptake, willingness, hesitancy, barriers, facilitators, and biomedical outcomes, but these findings are often reported separately. Less attention has been given to how barriers and opportunities align across socio-ecological levels, how willingness and uptake may diverge at the study level, and how genotype-specific and immunogenicity evidence can inform vaccination strategies for MSM.

Since HPV vaccination evidence relevant to MSM is not always reported in MSM-only samples, this review was centered on MSM while retaining studies that included MSM together with related male or broader sexual and gender minority populations when MSM-relevant findings could be extracted. This framing does not treat transgender women, non-binary people, or broader LGBTQ+ groups as male populations; rather, these groups were considered related sexual and gender minority populations when they were included alongside MSM or when findings were directly relevant to MSM-focused HPV vaccination delivery.

This review therefore aimed to synthesize evidence on HPV vaccination uptake, willingness or acceptability, vaccine hesitancy, barriers, opportunities, and vaccine-oriented outcomes among MSM, while incorporating MSM-relevant evidence from related sexual and gender minority populations when such findings could be extracted. Specifically, we sought to: (1) describe the characteristics of the included studies; (2) map multilevel barriers and opportunities using the socio-ecological model; (3) explore possible study-level patterns in reported willingness and uptake; and (4) summarize genotype-specific and immunogenicity findings relevant to early and catch-up vaccination strategies as supplementary biological context.

## 2. Methodology

### 2.1. Study Design and Eligibility Criteria

This systematic review was conducted and reported in accordance with the Preferred Reporting Items for Systematic Reviews and Meta-Analyses 2020 (PRISMA 2020) guidelines [9]. The review protocol was registered in PROSPERO (registration number: CRD420261343787). No separate review protocol was prepared and published.

The review aimed to synthesize evidence on HPV vaccination uptake, willingness or acceptability, vaccine hesitancy, barriers, facilitators, opportunities, and vaccine-oriented outcomes among MSM, while retaining studies that included MSM together with broader sexual and gender minority populations, such as transgender women, non-binary participants, or broader LGBTQ+ samples, when MSM-relevant findings could be extracted.

Given the heterogeneity across included studies in design, population characteristics, and outcome measures, meta-analysis was not performed. Findings were instead synthesized narratively and descriptively to summarize determinants, barriers, opportunities, and vaccine-oriented evidence relevant to MSM-focused HPV vaccination, with MSM as the key population of interest.

Studies were eligible if they were original research published in English or Chinese between 1 January 2010 and 31 December 2025 and reported HPV vaccination-related outcomes among MSM or among broader sexual and gender minority samples that included MSM, when MSM-specific or MSM-relevant findings could be extracted.

Outcomes of interest included vaccination uptake, willingness or acceptability, vaccine hesitancy, knowledge and attitudes, barriers and facilitators to vaccination, and relevant economic or biomedical outcomes, such as cost-effectiveness, antibody responses, or genotype-related findings. Quantitative, qualitative, mixed-methods, health economic, and other original biomedical studies relevant to MSM-focused HPV vaccination were included.

We excluded conference abstracts, unpublished manuscripts, policy reports without empirical data, systematic or narrative reviews, studies published in languages other than English or Chinese, studies focusing exclusively on female HPV vaccination, and studies without extractable MSM-specific or MSM-relevant findings.

### 2.2. Search Strategy and Study Selection

**Figure 1:**
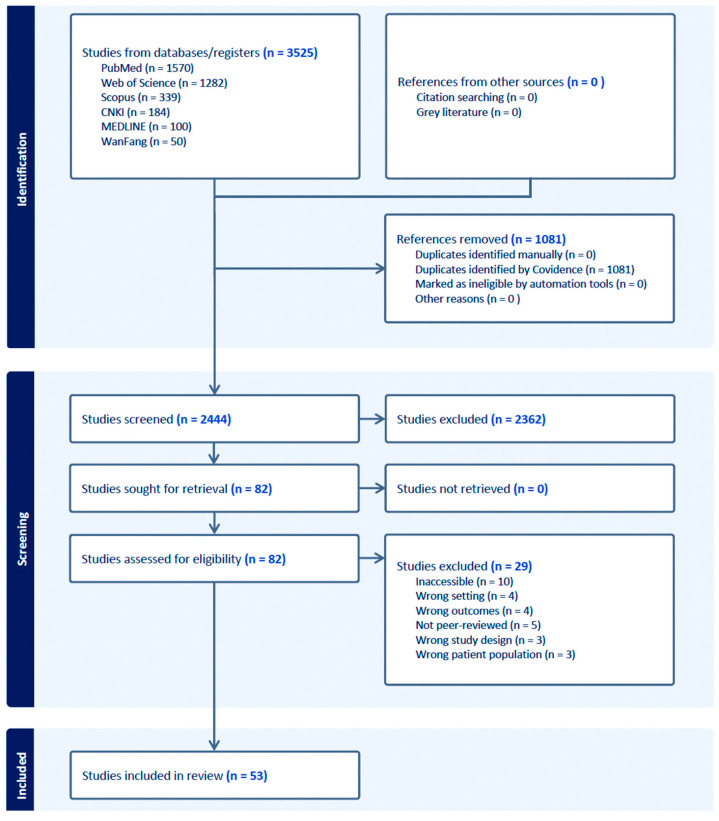
PRISMA flowchart for information search in PubMed, Web of Science, Scopus, CNKI, MEDLINE, and WanFang. The diagram shows the selection of reports included in the review.

The search strategy was developed by the research team, with contributions from trained research assistants and supporting team members involved in screening, data organization, and review coordination (Figure 1). Title and abstract screening (J.C., M.Z.) and full-text review (J.C., Z.L.) were conducted independently by trained reviewers, with disagreements resolved through discussion and consensus within the research team.

A comprehensive search was conducted across PubMed, Web of Science Core Collection, Scopus, Ovid MEDLINE, CNKI, and WanFang for studies published in English or Chinese between 1 January 2010 and 31 December 2025. The search strategy combined controlled vocabulary and free-text terms related to HPV vaccination and male populations. In the initial search stage, broad population terms such as “male” and “men” were used to maximize sensitivity and capture the full range of literature on HPV vaccination among males. The search was not restricted to MSM at this stage. In PubMed, for example, the search included the MeSH term “papillomavirus vaccines” together with title/abstract terms such as “Papillomavirus Vaccine,” “Human Papillomavirus Vaccine,” and “HPV Vaccine,” combined with “male” or “men.” Comparable adaptations of the search syntax were applied across the other databases according to their indexing systems and search interfaces. The complete database-specific search strategies are provided in Supplementary Table S1.

English and Chinese were not used as Boolean search terms. Language eligibility was applied using database language filters where available and otherwise during screening and full-text assessment. During full-text review, studies were retained when they reported MSM-specific findings or findings directly relevant to MSM-focused HPV vaccination implementation. Studies from broader male or sexual and gender minority samples were retained only when MSM-specific or MSM-relevant findings could be extracted.

All retrieved records were exported to Covidence for duplicate removal and systematic screening. The study selection process is summarized in the PRISMA flow diagram (Figure 1). A total of 3525 records were identified from six databases, including PubMed (*n* = 1570), Web of Science Core Collection (*n* = 1282), Scopus (*n* = 339), CNKI (*n* = 184), Ovid MEDLINE (*n* = 100), and WanFang (*n* = 50). No additional records were identified through citation searching or grey literature sources. After removal of 1081 duplicate records, 2444 records remained for title and abstract screening, of which 2362 were excluded.

Eighty-two reports were sought for retrieval, and all were successfully retrieved and assessed for eligibility. After applying the predefined inclusion and exclusion criteria, 29 reports were excluded for the following reasons: inaccessible full text (*n* = 10), wrong setting (*n* = 4), wrong outcomes (*n* = 4), not peer-reviewed (*n* = 5), wrong study design (*n* = 3), and wrong patient population (*n* = 3). Ultimately, 53 studies were included in the review and taken forward for data extraction and narrative synthesis.

### 2.3. Data Extraction

Data extraction was conducted using a standardized data extraction form developed by the research team. Extracted variables included general study characteristics, such as first author, publication year, manuscript title, DOI/PMID, journal, study objectives, study design, data collection methods, study setting, number of study sites, and data collection period (Supplementary Table S2). Geographic location was extracted as the study location reported in each included article and was used only for descriptive mapping and regional summary. Location categories were not intended to imply political, administrative, or sovereignty positions.

Population-related variables included sample size, eligibility criteria, population type, sex assigned at birth considerations, age range, ethnicity, HIV status, PrEP status, HPV infection history, HPV vaccination status, and other relevant demographic or behavioral characteristics. Population type was coded according to the participant groups reported in each study. Coding categories included MSM, GBM/GBMSM, transgender women (TGW), broader LGBTQ+ samples, mixed sexual and gender minority samples, and Other. “Mixed” was used when a study sample included MSM together with at least one other sexual or gender minority subgroup, such as transgender women, non-binary or gender-diverse participants, or broader LGBTQ+ participants.

To clarify how MSM populations were operationalized across studies, we additionally identified whether male sex assigned at birth was explicitly required for study eligibility. Studies were coded as “Yes” if eligibility criteria clearly required participants to be male or assigned male at birth. Studies were coded as “No” if eligibility was based on self-identified MSM, GBMSM, sexual behavior, or broader LGBTQ+ inclusion without explicitly requiring male sex assigned at birth. Studies were coded as “Not mentioned” when eligibility criteria did not provide sufficient information to determine whether sex assigned at birth was required.

Ethnicity categories were harmonized during data extraction. White included Caucasian, non-Hispanic White, or populations described as predominantly White; Black included Black, African, or African American populations; Hispanic/Latino included any population identified as Hispanic or Latino regardless of race; Asian included East Asian populations, such as Han or Chinese, and other Asian populations; Indigenous included Aboriginal, Torres Strait Islander, American Indian, or other Indigenous populations; Mixed/Multiracial included populations explicitly described as mixed or multiracial; and Other included ethnic groups not fitting the above categories or aggregated minor groups. Ethnicity was coded as Not Reported when ethnicity was not provided or when only nationality or country of birth was reported. When a population was described as highly homogeneous, such as >90% Han or White, it was classified under the corresponding category. When multiple ethnicities were reported, all categories were mapped into the harmonized categories.

HPV vaccination-related outcomes included actual vaccination coverage, vaccination willingness, vaccine hesitancy, vaccination status (fully vaccinated, partially vaccinated, or not vaccinated), and reported uptake of specific vaccine types, including 4-valent and 9-valent HPV vaccines. Additional subgroup analyses and subgroup-specific findings were collected where available.

Throughout the descriptive synthesis, uptake was defined as receipt of at least one HPV vaccine dose whenever this information was available. Partial vaccination and full-series completion were reported separately when distinguished in the original study. When only series completion or an unspecified vaccination measure was reported, the original study definition was retained.

Reported barriers and opportunities related to HPV vaccination were extracted for subsequent coding under the socio-ecological model. Opportunities were defined as factors reported by the included studies as facilitators of HPV vaccination, or as factors associated with higher uptake, willingness, or acceptability, access, or vaccination completion. Immunogenicity, antibody response, and genotype-related outcomes were extracted where reported.

Three reviewers (J.C., Z.L., and Y.Z.) independently extracted and cross-checked all study data to ensure consistency and accuracy. Any discrepancies identified during the extraction process were resolved through discussion and consensus within the research team. Methodological quality assessment was conducted for the included studies, with the assessment findings summarized in Section 3.

### 2.4. Narrative Synthesis and Socio-Ecological Model Analysis

Given the heterogeneity of study designs, populations, and outcome measures, findings were synthesized narratively and descriptively. Descriptive counts were used to summarize study-level reports of barriers and opportunities. These counts represent the number of studies reporting each theme and should not be interpreted as participant-level prevalence or effect estimates.

The socio-ecological model was used as the primary analytical framework to organize determinants of HPV vaccination among MSM [10]. This model conceptualizes health behavior as being shaped by interacting influences across multiple levels, including individual, interpersonal, organizational, community, and policy environments. In this review, extracted barriers and opportunities were coded into five domains: individual, provider-related interpersonal, organizational/institutional, community, and policy/societal levels.

The individual level captured participant-level knowledge, attitudes, beliefs, perceived risk, confidence, cost concerns, and intentions. The provider-related interpersonal level captured healthcare provider recommendation, communication, disclosure-related interactions, provider knowledge and engagement, stigma within healthcare interactions, LGBTQ+-affirming care, and provider-mediated access. The organizational/institutional level captured workforce training and capacity, service availability and delivery constraints, clinical workflow and system design, institutional practices, and healthcare infrastructure. The community level captured structural prevention context, stigma and social norms, knowledge and awareness environments, social networks and information flow, and community engagement. The policy/societal level captured vaccination policies, eligibility restrictions, insurance coverage, vaccine cost, and broader structural access factors. The SEM coding framework, subtheme working definitions, and domain allocation are provided in Supplementary Table S3.

The interpersonal level was operationalized specifically as provider-related interpersonal factors because the extractable interpersonal barriers and opportunities in the included studies primarily concerned healthcare provider recommendation, provider knowledge, patient–provider communication, disclosure, stigma within healthcare interactions, LGBTQ+-affirming care, and provider-mediated access to vaccination. Factors with similar meanings were grouped into broader thematic categories through iterative review and consensus among the research team. The frequency and continental distribution of identified themes were then summarized to identify recurring multilevel patterns.

SEM coding used a hybrid approach. The five SEM domains were applied deductively, based on the predefined socio-ecological framework, while subthemes within each domain were developed inductively from the extracted barrier and opportunity data. For barriers, initial coding was conducted by Z.L. using the extraction table, and J.C. subsequently checked each code against the extraction table and original articles. For opportunities, initial coding was conducted by Y.Z., and J.C. subsequently checked each code against the extraction table and original articles. Disagreements or uncertain coding decisions were resolved through discussion and consensus. Formal inter-rater agreement statistics were not calculated because coding was conducted through initial coding followed by verification and consensus rather than fully independent duplicate coding.

A single study could contribute more than one barrier or opportunity theme if it reported multiple relevant determinants. However, each study was counted only once for a given theme, regardless of how many times that theme appeared in the article. Barrier and opportunity themes were coded separately, and the resulting counts therefore represent the number of studies reporting each theme rather than the number of mentions, participants, or effect estimates. For the continental distribution of barriers and opportunities, the number of studies reporting each theme was normalized by the total number of studies from the corresponding region included in the barrier or opportunity mapping and expressed as a percentage. Percentages were calculated as (*n/N) × 100%*, where *n* represents the number of studies reporting the theme and *N* represents the total number of mapped studies from that region.

### 2.5. Clustering Analysis of HPV Vaccination Willingness and Uptake

An exploratory K-means analysis was conducted as a descriptive method to examine possible study-level patterns between reported HPV vaccination willingness and uptake among studies that provided both outcomes. Two variables were extracted from each eligible study: the reported HPV vaccination willingness rate and the reported vaccination uptake rate. For each study, uptake was extracted according to the review-defined criterion of receipt of at least one HPV vaccine dose when extractable, and willingness was extracted using the closest available study-level measure reported in the original article. The specific study-level definitions of uptake and willingness for all nine studies are provided in Supplementary Table S4. For each study, the input vector was defined as follows:

(1) $$x_{i}=\left(w_{i},v_{i}\right)$$

where $w_{i}$ represents the HPV vaccination willingness rate and $v_{i}$ represents the vaccination uptake rate for study i. Both variables were converted into proportions ranging from 0 to 1 before clustering. K-means clustering was applied to group studies with similar willingness–uptake profiles [11]. The algorithm partitions studies into K clusters by minimizing the within-cluster sum of squared distances:

(2) $$\min\sum_{k=1}^{K}\sum_{x_{i}\in C_{k}}{\left|\left|x_{i}-\mu_{k}\right|\right|}^{2}$$

where $C_{k}$ denotes the k-th cluster and $\mu_{k}$ denotes the centroid of that cluster. This objective assigns each study to the nearest centroid so that studies within the same cluster have similar willingness and uptake patterns. Candidate k values ranging from 2 to 7 were examined descriptively using average silhouette scores. The *k* = 4 solution had the highest average silhouette score [4]. However, because only nine studies were available, this solution was used solely to summarize possible study-level willingness–uptake patterns and was not considered evidence of a stable or validated cluster structure. For each study i, the silhouette score was calculated as follows:

(3) $$s\left(i\right)=\frac{b\left(i\right)-a\left(i\right)}{\max\left(a\left(i\right),b\left(i\right)\right)}$$

where a(i) is the average distance between study i and other studies in the same cluster, and b(i) is the smallest average distance between study i and studies in the nearest other cluster. A higher silhouette score indicates better cluster separation. The final number of clusters was selected as the k value with the highest average silhouette score.

For visualization, a crosshair divider plot was generated using the mean *x*- and *y*-coordinates of the final cluster centroids. These reference lines were used only to aid interpretation of relatively high or low willingness and uptake patterns and did not represent formal K-means classification boundaries. To further compare multilevel determinants across exploratory clusters, study-level matrix summaries were constructed to display the distribution of barrier and opportunity themes identified through SEM coding.

### 2.6. Quality Assessment

Methodological quality assessment was conducted for 51 of the included studies using study-design-specific appraisal tools. Cross-sectional, cohort and qualitative studies were assessed using the Joanna Briggs Institute (JBI) Critical Appraisal Checklists; randomized controlled trials were assessed using the Cochrane Risk of Bias 2 (RoB 2) tool, and economic evaluations were assessed using the Drummond Checklist for Economic Evaluations.

Two reviewers (Y.Z. and J.C.) independently assessed the methodological quality of 51 included studies using the appropriate study-design-specific appraisal tools. Discrepancies were resolved through discussion and consensus. When consensus could not be reached, a third reviewer (Z.L.) was consulted. The quality assessment was used to characterize the strength and limitations of the evidence base rather than as a criterion for study exclusion. Therefore, all eligible studies were retained regardless of methodological quality. The results of the quality assessment are reported in Section 3.2 and Supplementary Figures S1 and S2.

Since formal GRADE assessment was not feasible due to heterogeneity in study designs, populations, and outcomes and the absence of meta-analysis, we provided a concise narrative confidence summary based on study design, methodological quality, consistency of findings, directness to MSM HPV vaccination, geographic coverage, and outcome reporting.

## 3. Result

### 3.1. Study Selection and Characteristics of Included Studies

**Figure 2:**
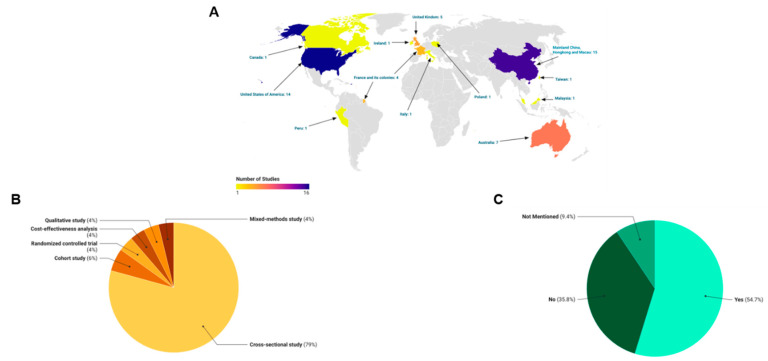
Characteristics of the 53 included studies in terms of geographic distribution, study design, and eligibility definitions. Panel (**A**). The geographic distribution of the included studies is shown by country or territory, using a thematic world map that indicates the number of studies conducted in each location. The map, created with Datawrapper, is based on 52 studies for which geographic information could be extracted; one study involving a global, multi-continent trial was not tied to a specific location and was therefore omitted from the map. A color gradient from yellow to purple is used, where yellow represents the fewest studies and purple the most. For mapping purposes, study locations were recorded and displayed according to the geographic extraction and visualization categories used in this review. Mainland China, Hong Kong, Macao, and Taiwan were categorized as study-location entries according to the extraction framework. Studies conducted in France, as well as French overseas territories and departments where applicable, were grouped according to the location categories reported in the included studies. These categories were used solely for descriptive mapping and should not be interpreted as statements regarding political or administrative status. Panel (**B**). Distribution of study designs among the included studies. Proportional representation of study designs included in this systematic review of HPV vaccination among men who have sex with men (*n* = 53). Panel (**C**). Distribution of sex-at-birth criteria within MSM eligibility definitions across the included studies. The pie chart illustrates whether studies explicitly required participants to be male at birth as part of their definition of MSM eligibility. Studies were classified into three categories: “Yes” (male sex at birth explicitly required), “No” (this criterion was not applied), and “Not mentioned” (the eligibility criteria did not clearly state whether sex assigned at birth was required).

Following the study selection process summarized in Figure 1, 53 studies were included. Study locations were geographically unevenly distributed. The largest number of studies was conducted in locations categorized as Mainland China, Hong Kong, and Macao in our geographic extraction framework (*n* = 15), followed by the United States (*n* = 14), Australia (*n* = 7), and the United Kingdom (*n* = 5) (Figure 2A). Additional study locations included France, French overseas territories and departments where applicable, Canada, Ireland, Italy, Poland, Peru, Taiwan, and Malaysia, indicating broad but geographically concentrated evidence.

The studies used several methodological approaches (Figure 2B). Cross-sectional studies predominated (79%), while randomized controlled trials, cohort studies, qualitative studies, mixed-methods studies, and cost-effectiveness analyses were less common. Thus, most evidence described uptake, willingness, and barriers at a single time point, with comparatively limited longitudinal, intervention, qualitative, and economic data.

Across 52 studies with usable sample-size data, 169,241 participants were included. MSM accounted for 147,566 participants (87.2%), while the remaining 21,675 participants (12.8%) came from other eligible samples with extractable MSM-relevant or male-specific findings, giving an MSM-to-non-MSM ratio of approximately 6.81:1 (Table 1). Eligibility criteria based on sex assigned at birth varied (Figure 2C). Slightly more than half of the studies explicitly required participants to be male or assigned male at birth (54.7%); 35.8% did not apply this criterion, and 9.4% did not report it clearly.

Ethnicity was reported in 32 of 53 studies (60.4%); 21 studies (39.6%) did not report ethnicity-related data. Because ethnicity was extracted at the study level, the categories indicate the number of studies reporting each group rather than the pooled participant ethnicity. White ethnicity was reported in 19 studies and Asian ethnicity in 17 studies; Black, Hispanic/Latino, Indigenous, Mixed/Multiracial, and Other categories were reported less often (Table 1).

### 3.2. Methodological Quality Assessment

Methodological quality assessment was completed for 51 included studies using study-design-specific appraisal tools (Supplementary Figures S1 and S2). Overall, the methodological quality of the included evidence was considered acceptable, although quality varied across study designs.

Among the studies assessed using JBI-based tools, most were rated as having low or unclear risk of bias, with only a small number classified as high risk of bias. The two randomized controlled trials assessed using RoB 2 were judged to have some concerns, primarily related to potential sources of bias in the randomization or outcome assessment process. The two economic evaluation studies assessed using the Drummond Checklist generally demonstrated adequate reporting quality, although limitations in reporting assumptions and study limitations were identified.

Across study designs, most studies clearly reported their objectives, study populations, and HPV vaccination-related outcomes. Common methodological limitations included insufficient control of potential confounding factors, incomplete reporting of participant selection procedures, limited discussion of sources of uncertainty, and inadequate reporting of study limitations. The quality assessment was used to contextualize the strength of the evidence rather than to determine study exclusion. Accordingly, the findings of this review should be interpreted as study-level descriptive evidence rather than participant-level prevalence estimates or pooled causal effects.

### 3.3. Continental Distribution of Barriers to HPV Vaccination Across the Socio-Ecological Model

**Figure 3:**
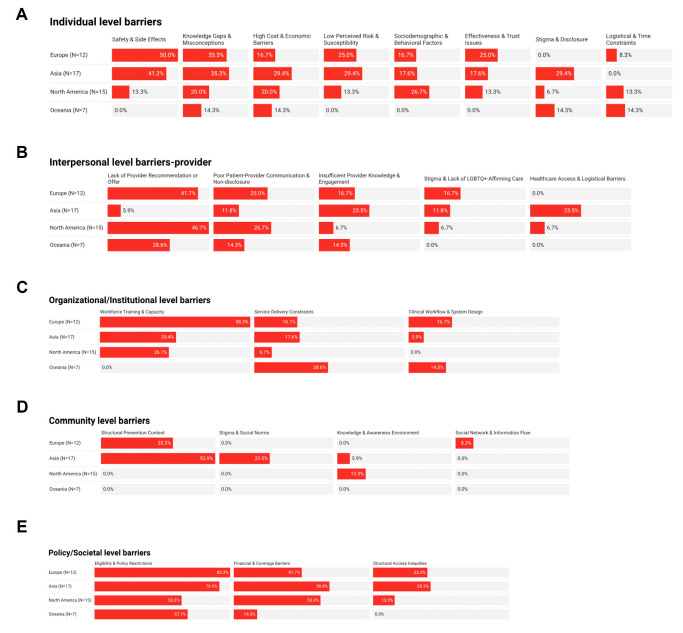
Socio-ecological model-based continental distribution of barriers to HPV vaccination. Barriers were classified using a socio-ecological framework and organized into five levels: individual, interpersonal, organizational/institutional, community, and policy/societal. In this review, the interpersonal level specifically refers to provider-related barriers. Panel (**A**) presents individual-level barriers; Panel (**B**), provider-related interpersonal barriers; Panel (**C**), organizational/institutional barriers; Panel (**D**), community-level barriers; and Panel (**E**), policy/societal barriers. The bars represent the percentage of studies within each region reporting each barrier theme. Percentages were calculated using the number of studies reporting the theme as the numerator and the total number of studies from that region included in the barrier mapping as the denominator: Europe (*n* = 12), Asia (*n* = 17), North America (*n* = 15), and Oceania (*n* = 7). These percentages reflect study-level reporting frequencies rather than participant-level prevalence. Values are rounded to one decimal place. Overall study-level counts reported in the text were obtained by summing the corresponding underlying regional counts. Two studies that examined biological or clinical HPV outcomes, specifically anal HPV genotype prevalence and HPV antibody/immunogenicity, were excluded from the barrier mapping (see Supplementary Table S2 extraction IDs 282 and 285).

Using the socio-ecological framework, barriers were distributed across all five levels and varied by continent (Figure 3). Individual-level barriers generated the largest number of study-theme reports (*n* = 78), followed by policy/societal barriers (*n* = 71), provider-related interpersonal barriers (*n* = 43), organizational/institutional barriers (*n* = 28), and community-level barriers (*n* = 21). To account for differences in the number of included studies across regions, Figure 3 presents each barrier theme as the percentage of mapped studies within the corresponding region that reported that theme. Therefore, regional patterns should be interpreted as normalized study-level reporting frequencies rather than true regional burden or prevalence. To account for differences in the number of mapped studies across regions, Figure 3 presents the percentage of studies within each region reporting each barrier theme. Regional patterns are therefore interpreted as normalized study-level reporting frequencies rather than as population-level burden or prevalence.

At the individual level, safety and side-effect concerns (*n* = 15), knowledge gaps and misconceptions (*n* = 14), high cost and economic barriers (*n* = 11), and low perceived risk or susceptibility (*n* = 10) were the most frequently reported themes. In absolute study-level counts, Asian studies reported the largest number and widest range of individual-level barrier themes. In European studies, safety, knowledge, and trust-related barriers were commonly reported, whereas North American studies reported a more even distribution across knowledge gaps, cost, sociodemographic or behavioral factors, logistical constraints, and trust-related issues (Figure 3A).

At the provider-related interpersonal level, lack of provider recommendation or offer was the most frequent theme (*n* = 15), followed by poor patient–provider communication and non-disclosure (*n* = 10), insufficient provider knowledge and engagement (*n* = 8), stigma/discrimination and limited LGBTQ+-affirming care (*n* = 5), and healthcare access or logistical barriers (*n* = 5). In absolute study-level counts, lack of provider recommendation was reported most often in studies from North America and Europe, whereas Asian studies more often reported insufficient provider knowledge, stigma or limited LGBTQ+-affirming care, and healthcare access or logistical constraints (Figure 3B).

At the organizational/institutional level, workforce training and capacity were the most frequently reported barrier theme (*n* = 16), followed by service delivery constraints (*n* = 8) and clinical workflow and system design barriers (*n* = 4). After regional normalization, workforce training and capacity were most frequently reported among European studies, whereas service delivery constraints were reported across all four regions. Clinical workflow and system design barriers were reported in Europe, Asia, and Oceania (Figure 3C). At the community level, the structural prevention context was the most frequently reported barrier theme (*n* = 13), followed by stigma and social norms (*n* = 4), knowledge and awareness of the environment (*n* = 3), and social network and information flow (*n* = 1). Structural prevention context was reported in Asian and European studies, while stigma and social norms were reported in Asian studies. Knowledge and awareness of the environment were reported in North American and Asian studies, and social network and information flow were reported in one European study (Figure 3D). At the policy/societal level, eligibility and policy restrictions were most frequent (*n* = 35), followed by financial and coverage barriers (*n* = 24) and structural access inequities (*n* = 12) (Figure 3E).

### 3.4. Continental Distribution of Opportunities to Enhance HPV Vaccination Across the Socio-Ecological Model

**Figure 4:**
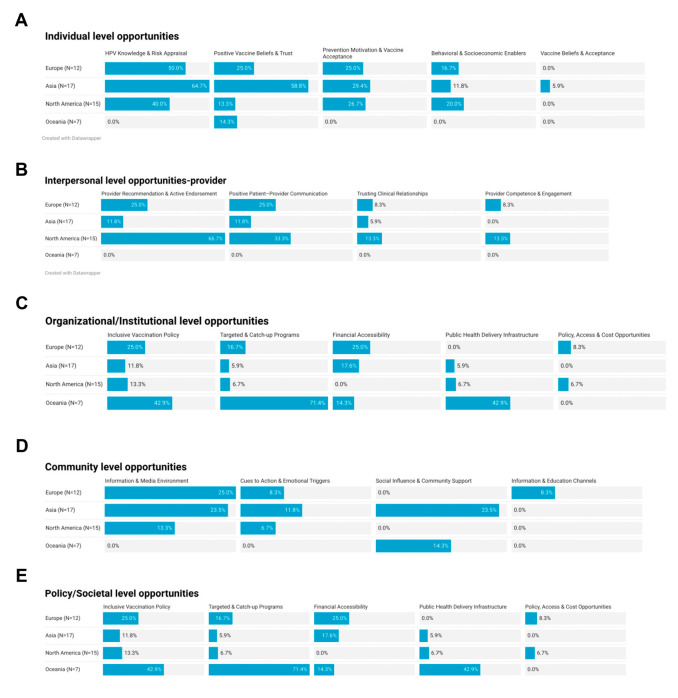
Socio-ecological model-based continental distribution of opportunities to enhance HPV vaccination. Opportunities were categorized using the same socio-ecological framework and divided into five levels: individual, interpersonal, organizational/institutional, community, and policy/societal. Panel (**A**) displays individual-level opportunities; Panel (**B**), provider-related interpersonal opportunities; Panel (**C**), organizational/institutional opportunities; Panel (**D**), community-level opportunities; and Panel (**E**), policy/societal opportunities. The bars represent the percentage of mapped studies within each region reporting each opportunity theme. Percentages were calculated using the number of studies reporting the theme as the numerator and the total number of studies from that region included in the opportunity mapping as the denominator: Europe (*n* = 12), Asia (*n* = 17), North America (*n* = 15), and Oceania (*n* = 7). These percentages reflect study-level reporting frequencies rather than participant-level prevalence. Values are rounded to one decimal place. Overall study-level counts reported in the text were obtained by summing the corresponding underlying regional counts. Within each SEM level, subthemes are ordered from left to right in descending order according to the total number of study-level reports summed across Europe, Asia, North America, and Oceania. Two studies that examined biological or clinical HPV outcomes, specifically anal HPV genotype prevalence and HPV antibody/immunogenicity, were excluded from the opportunity mapping (see Supplementary Table S2, extraction IDs 282 and 285).

Opportunities to enhance HPV vaccination were mapped across the same five levels: individual, provider-related interpersonal, organizational/institutional, community, and policy/societal (Figure 4). Individual-level opportunities generated the largest number of study-level reports (*n* = 59), followed by organizational/institutional (*n* = 41), policy/societal (*n* = 33), provider-related interpersonal (*n* = 32), and community-level opportunities (*n* = 19). As with barriers, these counts reflect study-level coding frequencies rather than pooled effect sizes or participant-level prevalence. To account for regional differences in the number of studies included, Figure 4 presents each opportunity theme as the percentage of mapped studies within the corresponding region that reported that theme. Regional differences in opportunity themes were therefore interpreted as normalized study-level reporting patterns, not as population-level prevalence of facilitators.

At the individual level, HPV knowledge and risk appraisal were the most frequent opportunity theme (*n* = 23), followed by positive vaccine beliefs and trust (*n* = 16), prevention motivation and vaccine acceptance (*n* = 12), and behavioral or socioeconomic enablers (*n* = 7). In absolute study-level counts, these opportunities were reported most often in Asian studies, while Europe and North America also showed recurring reports related to HPV knowledge and risk appraisal (Figure 4A).

Provider-related opportunities were centered on provider recommendation and active endorsement (*n* = 15), positive patient–provider communication (*n* = 10), trusting clinical relationships (*n* = 4), and provider competence and engagement (*n* = 3). In absolute study-level counts, North American studies contributed the largest number of provider-related opportunity reports, particularly for provider recommendation and communication (Figure 4B).

Organizational/institutional opportunities were concentrated around integrated service delivery (*n* = 17), accessible and convenient vaccination services (*n* = 15), and proactive vaccination infrastructure (*n* = 9). These themes were most often reported in European and North American studies in absolute study-level counts but were also reported in Asia and Oceania (Figure 4C). Community-level opportunities were less frequent and included information and media environment (*n* = 9), social influence and community support (*n* = 5), cues to action and emotional triggers (*n* = 4), and information and education channels (*n* = 1), with the largest absolute number of community-level study reports from Asian studies (Figure 4D).

At the policy/societal level, the main opportunity themes were inclusive vaccination policy (*n* = 10), targeted and catch-up programs (*n* = 9), financial accessibility (*n* = 7), public health delivery infrastructure (*n* = 5), and broader policy, access, and cost opportunities (*n* = 2). Policy-level opportunity reports from Oceania should be interpreted cautiously because they were based on a smaller number of included studies. Europe and Asia showed recurring opportunity reports related to inclusive policy and financial accessibility (Figure 4E).

### 3.5. Exploratory Clustering Analysis of HPV Vaccination Uptake and Willingness

**Figure 5:**
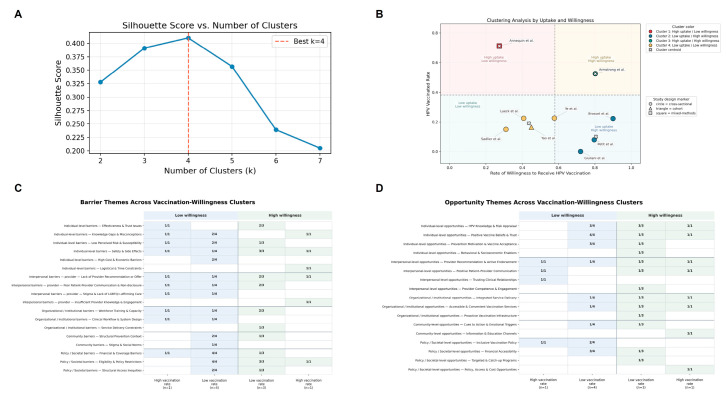
Exploratory clustering analysis of HPV vaccination uptake, willingness, and study-level profiles of barriers and opportunities. Panel (**A**). Silhouette analysis is used to determine the optimal number of clusters. Silhouette scores were assessed across various candidate *k* values, with the highest score observed at *k* = 4. Panel (**B**). Clustering plot of HPV vaccination uptake and willingness among nine studies with complete data on both outcomes. Each point represents a study, with the x-axis showing the reported willingness to receive HPV vaccination and the y-axis showing reported HPV vaccination uptake. Study labels indicate the first author of each of the nine included studies; detailed study information is provided in Supplementary Table S4. Point colors indicate the four exploratory uptake–willingness profiles: high uptake/low willingness, low uptake/low willingness, low uptake/high willingness, and high uptake/high willingness. Point shapes indicate study design, and white cross markers indicate cluster centroids. The dashed vertical and horizontal lines are visual reference lines separating relatively higher and lower willingness and uptake and should not be interpreted as formal classification thresholds. Panels (**C**,**D**). Study-level matrix summaries of barrier themes and opportunity themes, respectively, across the four uptake–willingness profiles. Cell values indicate the number of studies within a given profile that reported the corresponding theme, divided by the total number of studies in that profile. Blue-shaded cells correspond to low-willingness profiles, and green-shaded cells correspond to high-willingness profiles. Empty cells indicate that no study in that profile reported the corresponding theme. Given the nine-study sample and the presence of single-study groups, Panels (**C**,**D**) should be interpreted as descriptive summaries rather than statistical comparisons.

Nine studies reported complete data on both HPV vaccination uptake and willingness and were included in the exploratory clustering analysis. Among the candidate *k* values from 2 to 7, *k* = 4 had the highest average silhouette score, with scores of 0.3278 (*k* = 2), 0.3910 (*k* = 3), 0.4101 (*k* = 4), 0.3569 (*k* = 5), 0.2393 (*k* = 6), and 0.2055 (*k* = 7) (Figure 5A). Although the separation was moderate, the four-group solution provided a descriptive summary of possible study-level willingness–uptake patterns (Figure 5B).

The four-group solution comprised high uptake/low willingness, low uptake/low willingness, low uptake/high willingness, and high uptake/high willingness (Figure 5B; Table 2). The two high-uptake groups each contained one study and were interpreted descriptively. Among the seven studies in the low-uptake groups, three showed relatively high willingness and four showed low willingness. The low uptake/high willingness group may indicate a possible gap between reported willingness and vaccination uptake, whereas the low uptake/low willingness cluster may reflect both limited vaccination intention and low uptake.

The crosshair divider plot visualized the four willingness–uptake regions (Figure 5B), with reference lines at approximately *x* = 0.579 and *y* = 0.382. These lines distinguish relatively higher or lower willingness and uptake, while K-means clustering determined cluster assignment. Seven of the nine studies fell into low-uptake clusters, distinguishing two forms of low vaccination coverage: low uptake with low willingness and low uptake despite high willingness.

The barrier matrix summarized the study-level distribution of reported barrier themes across the four exploratory uptake–willingness clusters (Figure 5C). In the low uptake/low willingness cluster, eligibility and policy restrictions and financial and coverage barriers were reported in all four studies. These structural barriers co-occurred with individual-level barriers, including knowledge gaps, low perceived risk, and high cost, each reported in two studies. This pattern suggests the coexistence of limited perceived need, insufficient knowledge, affordability constraints, and policy restrictions. In the low uptake/high willingness cluster, all three studies reported safety and side-effect concerns and eligibility or policy restrictions. Two of the three studies also reported effectiveness or trust issues, lack of provider recommendation, poor patient–provider communication, and limitations in workforce training and capacity. This co-occurrence suggests that relatively high willingness may not translate into uptake when safety concerns, limited provider support, workforce capacity limitations, and restrictive access conditions remain.

The study-level matrix summaries of opportunities further characterized the clusters (Figure 5D). In the low uptake/low willingness cluster, opportunity themes centered on demand generation and affordability: vaccine beliefs and acceptance were reported in all four studies, and HPV knowledge and risk appraisal, prevention motivation, and financial accessibility were each reported in three. In the low uptake/high willingness cluster, HPV knowledge and risk appraisal appeared in all three studies, whereas affordability, provider/system intervention, and policy-related opportunities were less consistent. High-uptake clusters showed provider/system, policy, information, and access-related opportunity themes, but each contained one study, so these findings should be interpreted as descriptive signals rather than stable cluster-level patterns.

### 3.6. Genotype-Specific and Vaccine-Oriented Findings

Two included studies examined biological or clinical HPV outcomes rather than SEM-coded barriers or opportunities and were therefore summarized separately [12,13]. In a targeted catch-up setting of 496 previously unvaccinated MSM aged 20–26 years attending a Melbourne sexual health clinic before their first HPV vaccine dose, 75.2% had at least one anal HPV genotype, 56.5% had at least one high-risk genotype, 43.1% had at least one quadrivalent vaccine-targeted genotype, and 53.4% had at least one nonavalent vaccine-targeted genotype; HPV16 was detected in 21.0% of participants [13]. These findings suggest that although prior HPV exposure was common in this clinic-based population, many participants had not acquired all vaccine-targeted genotypes, indicating a potential role for catch-up vaccination.

In a separate study comparing 200 unvaccinated and 127 vaccinated young MSM, median antibody levels were higher among vaccinated participants than among those with presumed natural infection for HPV6, HPV11, HPV16, and HPV18, with levels of 223 versus 48 mMU/mL, 163 versus 21 mMU/mL, 888 versus 72 mMU/mL, and 161 versus 20 mMU/mL, respectively. Antibody levels among vaccinated participants appeared stable for up to 66 months [12]. These findings are consistent with a robust and sustained vaccine-induced antibody response, although clinical protection was not directly assessed. Taken together, the two studies provide limited biological support for vaccination before exposure and suggest potential benefit from catch-up vaccination.

## 4. Discussion

### 4.1. Principal Findings and Interpretation

This review shows that HPV vaccination among MSM and related MSM-relevant populations is shaped by interacting factors across multiple socio-ecological levels rather than by individual willingness alone. A central finding is that many barriers had corresponding opportunities within the same socio-ecological level, suggesting that the same implementation pathways may either constrain or enable vaccination depending on whether trusted information, supportive provider communication, accessible services, community engagement, and inclusive policy are present (Table 3). Accordingly, we interpret the paired barrier–opportunity themes as modifiable implementation pathways rather than as separate lists of problems and solutions.

This socio-ecological interpretation is consistent with broader vaccine uptake in the literature. Social ecological frameworks have been used to organize barriers and facilitators of COVID-19 vaccine acceptance across intrapersonal, interpersonal, institutional, community, and public policy levels, showing that vaccine behavior is shaped by both individual concerns and wider access, trust, and policy conditions [14]. Similar multi-level approaches have also been applied to childhood vaccination demand and to HPV vaccination decision-making among young women, where family, provider, organizational, community, and policy-level influences shaped vaccine uptake [15,16]. These studies support the value of interpreting HPV vaccination among MSM as a multi-level implementation issue rather than as a matter of individual willingness alone.

At the individual level, the findings suggest that knowledge and trust are necessary but insufficient conditions for vaccination. Education for MSM should therefore move beyond general awareness of HPV and address anal, penile, and oropharyngeal cancer risks, the value of vaccination before HPV exposure, and the potential benefit of catch-up vaccination for those who may not have encountered all vaccine-preventable genotypes. Importantly, willingness may coexist with unresolved concerns about safety, effectiveness, cost, eligibility, and where to receive the vaccine; therefore, information-based interventions are unlikely to be sufficient unless they are paired with trusted communication and accessible services.

These individual-level findings are consistent with the broader HPV vaccination literature, particularly studies of adolescents, young women, and young adults. In a systematic review of barriers to HPV vaccination among U.S. adolescents, Holman et al. identified insufficient information, low perceived HPV risk, parental concerns, social influences, irregular preventive care, vaccine cost, and provider-related barriers as recurring constraints on vaccine uptake [17]. Similarly, a qualitative systematic review of HPV vaccination among young women found that vaccination decisions were shaped not only by individual beliefs, but also by policymakers, healthcare professionals, parents, financial considerations, social norms around sexuality, and trust in vaccination programmes and providers [16]. A more recent systematic review of HPV vaccination among people aged 9–26 years also identified limited HPV and vaccine knowledge, concerns about vaccine safety and efficacy, cost, discrimination, perceived risk and benefit, and recommendations from others as important barriers or facilitators [18]. Together, these studies suggest that the knowledge gaps, safety concerns, cost barriers, provider influence, and missed opportunities observed among MSM are not unique to MSM but reflect broader challenges in HPV vaccine delivery.

The provider-related findings are central to this interpretation. Rather than functioning only as a source of information, provider recommendations may act as a key cue to action that helps convert willingness into vaccination. How providers frame HPV vaccination, deliver risk information, respond to concerns, and normalize vaccination can strongly influence the final uptake outcome. Evidence from broader HPV vaccination literature supports this point. Provider communication quality, strong endorsement, same-day recommendation, and cancer-prevention framing have been associated with higher HPV vaccine initiation and completion [19,20]. For MSM, provider recommendations also need to be confidential, non-judgmental, and inclusive. Poor patient–provider communication and non-disclosure were paired with trusting clinical relationships and disclosure, showing that communication quality affects whether patients feel safe enough to discuss sexual health, disclose risk, and accept vaccination.

However, MSM-focused HPV vaccination also involves barriers that are less prominent in adolescent or female-focused HPV vaccination literature. In a UK study of MSM, Nadarzynski et al. found that many MSM would accept HPV vaccination if it was recommended by healthcare professionals, but that optimal uptake would require reaching MSM who do not attend sexual health clinics and those unwilling to disclose their sexual orientation [21]. This suggests that provider recommendations may be especially important for MSM, while also being constrained by whether MSM are connected to sexual health services and feel safe discussing sexual behavior. A qualitative study among MSM in a medically underserved U.S. region similarly identified limited awareness, reliance on mainstream healthcare providers for vaccine information, stigma and reluctance to disclose sexual orientation, uncertainty about insurance coverage and vaccine costs, and distance or time required to access vaccination as key barriers [22]. These findings indicate that MSM-specific implementation barriers often arise at the intersection of provider communication, disclosure safety, service accessibility, and targeted eligibility or funding rules.

Organizational factors may help explain why willingness does not always translate into vaccination. For individuals who already report willingness, the priority is not only to persuade them but to remove friction between intention and vaccination. Practical strategies include offering vaccination during sexual health, HIV, PrEP, STI, and primary-care visits; using electronic or text reminders; applying standing orders or prompts within electronic health records; and reducing missed opportunities during routine clinical encounters. Evidence from intervention studies shows that reminders combined with provider recommendation can increase HPV vaccination, and multi-level interventions involving staff training, patient education, and reminders can reduce missed opportunities [23,24]. These approaches are particularly relevant for low-uptake/high-willingness contexts, where the main challenge is conversion from willingness to completed vaccination.

Community-level findings show why clinical and organizational changes may not be sufficient on their own. Some MSM may be highly connected to sexual health information, PrEP use, and regular testing, while others may avoid traditional healthcare settings because of stigma, fear of disclosure, or previous negative experiences. Community-led and peer-informed approaches may help reach these subgroups by creating safer spaces, meeting people where they are, and using messengers who understand lived experience. Community-led HIV responses are widely recognized as important for reducing stigma, improving outreach, and supporting prevention and treatment education among key populations [25]. Programme examples from Singapore, such as Action for AIDS MSM outreach, also illustrate how community education, targeted outreach, and testing-related services can be linked to broader sexual health engagement [26]. Although these examples come mainly from HIV and STI programmes, they offer useful lessons for HPV vaccination among MSM.

At the policy level, universal routine vaccination and targeted MSM catch-up should be understood as complementary but distinct strategies. Universal, gender-neutral routine vaccination should provide protection to adolescents before sexual debut and before disclosure of sexual orientation becomes relevant. Targeted catch-up should address MSM and other eligible adults who were not reached through routine vaccination, including through sexual health, HIV, PrEP, STI, primary-care, and community-linked services. For adults beyond routine catch-up ages, shared clinical decision-making or targeted access may remain appropriate according to local recommendations [27,28,29,30]. Programmes delivered through sexual health and HIV clinics, such as the UK model, illustrate how targeted MSM catch-up can address gaps left by routine vaccination rather than replace universal adolescent vaccination [31].

The two biological studies provide supplementary context for vaccination before exposure and possible catch-up benefit, but they do not establish clinical or programme effectiveness.

The exploratory clustering analysis should also be interpreted in light of differences in outcome measurement across studies. Although uptake was harmonized in this review as receipt of at least one HPV vaccine dose, willingness was less uniform because the available indicators reflected different forms of willingness, acceptability, intention, vaccine acceptance, or hesitancy-related constructs. Therefore, the clustering results should be understood as descriptive study-level patterns. However, the exploratory clustering findings may help generate hypotheses about different implementation needs, even if they should not be interpreted as stable programme typologies. For low-uptake/low-willingness settings, interventions may need to combine demand generation with structural reform. Education, risk appraisal, and trust-building are likely to be important, but they are unlikely to be sufficient if eligibility restrictions, cost, or limited-service availability remain unresolved. For low-uptake/high-willingness settings, the intervention logic may be different: the main task is to make vaccination easier, more immediate, and more trusted. This may involve same-day vaccination, reminders, provider prompts, clear navigation from online information to clinics, community referral pathways, and cost removal. For both clusters, safety concerns should be addressed directly, but not in a fear-based manner. A more effective approach is to frame HPV vaccination as routine cancer prevention, acknowledge concerns, provide simple and evidence-based reassurance, and offer a clear next step.

Taken together, these findings suggest an age- and setting-specific implementation pathway. For adolescents, the included evidence consistently supports the value of routine vaccination before sexual debut. For young adults and MSM who missed routine vaccination, studies highlighted the importance of catch-up strategies delivered through settings where sexual health services are already provided, including HIV, PrEP, STIs, and primary-care services. For adults beyond routine catch-up ages, the evidence suggests that provider engagement and individualized discussions may facilitate vaccination decisions among those with ongoing or future exposure risk. Rather than supporting a single implementation approach, the findings indicate that effective HPV vaccination strategies are likely to require coordinated action across individual, provider, organizational, community, and policy levels, with priorities varying according to the target population and healthcare context.

### 4.2. Strengths and Limitations

This review has several strengths. First, it synthesizes evidence from the broad international literature and organizes findings using a socio-ecological framework. Second, it maps both barriers and opportunities, which allows a more action-oriented interpretation than a barrier-only review. In addition, the paired barrier–opportunity table provides a concise structure for discussing how the same mechanisms can operate in opposite directions. Fourth, the exploratory clustering analysis provides a preliminary descriptive comparison between studies reporting low uptake with low willingness and those reporting low uptake despite relatively high willingness. Finally, the separate synthesis of genotype-specific and immunogenicity studies links behavioural and structural findings with vaccine biology.

Despite its contributions, several caveats must be acknowledged. First, the synthesis was based on study-level rather than participant-level evidence. In the thematic counting and clustering analysis, each study was treated as one evidence unit, without weighting by study sample size or by the estimated size of local or national MSM populations. Therefore, the distribution of barriers and opportunities reflects patterns in the published literature rather than population-level prevalence. This limitation is particularly important because the evidence base was geographically uneven, with relatively limited MSM-specific evidence from Africa, South America, and other underrepresented settings.

Second, the bilingual English–Chinese search strategy was both a strength and a limitation. Including Chinese-language databases allowed us to capture evidence that may be missed by English-only reviews. However, restricting eligibility to English- and Chinese-language publications may have undercaptured relevant studies published in other languages, such as Spanish, Portuguese, or French. In addition, the use of China- or Chinese-specific terms in selected searches may have increased retrieval from Chinese-language and China-related research contexts, contributing to the concentration of included studies from China and English-language publication settings.

Third, the exploratory study-level clustering analysis was constrained by the reporting and measurement approaches of the primary studies. Although 53 studies were included in the review, only nine reported quantitative data on both HPV vaccination uptake and willingness. In addition, complete harmonization was not possible because willingness, acceptability, and intention were defined differently across studies, even though uptake was harmonized to receipt of at least one HPV vaccine dose. These differences limit direct cross-study comparability and may affect the stability of the K-means solution. Study-specific definitions are provided in Supplementary Table S4.

Fourth, the genotype-specific immunogenicity synthesis was based on only two studies with distinct designs, populations, and outcomes. These findings should therefore be interpreted as descriptive biological context rather than definitive evidence of vaccine effectiveness, duration of clinical protection, or the effectiveness of catch-up vaccination programmes.

Finally, heterogeneity in population definitions and study designs may limit interpretation. Included studies varied in whether they reported sex assigned at birth, gender identity, and sexual behavior, which may obscure differences among cisgender MSM, transgender women, non-binary people, and broader sexual and gender minority populations. Most included studies were cross-sectional and many relied on self-reported vaccination status, limiting causal interpretation and introducing possible recall or social desirability bias. Studies conducted during or shortly after the COVID-19 pandemic may also reflect temporary disruptions in vaccine supply, clinic accessibility, or preventive healthcare use. Selective reporting in the primary literature may further mean that prominent or statistically significant barriers were more likely to be reported than less visible factors.

Overall, we have moderate confidence in the consistency of the identified implementation themes, as similar barriers and opportunities were reported across multiple countries and healthcare settings. However, confidence in the strength of individual associations remains limited because most included studies were cross-sectional, heterogeneous in design, and synthesized narratively without quantitative pooling. Therefore, the findings should be interpreted primarily as descriptive evidence to inform implementation and future research rather than as evidence of causal effects.

### 4.3. Implications for Research and Practice

Future research should move beyond documenting barriers and test how paired opportunities can be operationalized. Studies should evaluate whether provider training, strong recommendation scripts, reminders, same-day vaccination, community referral pathways, mobile services, and cost removal improve HPV vaccine initiation and completion among MSM. Future studies should also report uptake and willingness within the same analytic sample and at clearly defined assessment points, using explicit denominators and standardized outcome definitions, to support more reliable cross-study comparisons. More studies are needed in underrepresented regions and among diverse MSM subgroups and related sexual and gender minority populations, including transgender and gender-diverse people, migrants, adolescents before sexual debut, and adults who may benefit from shared decision-making. Future reporting should clearly define eligibility criteria, sex assigned at birth where relevant, gender identity, sexual behaviour, vaccination status, and vaccine schedule, so that findings can be compared across settings.

In practice, public health programmes should combine universal adolescent vaccination with targeted catch-up for MSM who were not reached through routine vaccination. Implementation should address barriers across multiple levels by improving HPV knowledge and vaccine confidence, strengthening inclusive provider recommendations, reducing missed opportunities in clinical settings, supporting peer- and community-linked outreach, and ensuring inclusive eligibility and financial coverage. The central implication of the paired SEM is that improving HPV vaccination among MSM requires changing the conditions around vaccination, not simply changing individual attitudes.

## 5. Conclusions

HPV vaccination among MSM is shaped by interacting determinants across all levels of the socio-ecological model. The repeated pairing of barriers with opportunities indicates that the same implementation pathways can either constrain or facilitate vaccination, depending on whether trusted information, provider support, accessible services, community support, and inclusive policy are present. The exploratory clustering analysis suggests potential differences between settings that may require both demand generation and structural reform and those in which relatively high willingness may not translate into vaccination uptake. Overall, the implementation findings are consistent with universal, gender-neutral routine adolescent vaccination and targeted catch-up for MSM who missed routine vaccination, delivered through trusted, convenient, and community-linked services. The two genotype-specific and immunogenicity studies provide supplementary biological context for vaccination before exposure and possible catch-up benefit, but do not independently establish clinical or programme effectiveness. Future studies should examine these implementation pathways using consistent definitions of uptake and willingness, prospective study designs, and more diverse MSM populations and healthcare settings.

## Figures and Tables

**Table 1:** Study Population Composition and Ethnicity Reporting Across Included Studies.

Characteristic	Total (*n*)	Percentage	% of Studies Reported Ethnicity
Study population composition			
MSM	147,566	87.2%	
Non-MSM	21,675	12.8%	
Total sample size	169,241	100%	
MSM:non-MSM ratio	6.81:1		
Ethnicity reporting across included studies			
White	19	35.8%	59.4%
Asian	17	32.1%	53.1%
Black	14	26.4%	43.8%
Hispanic/Latino	13	24.5%	40.6%
Mixed/Multiracial	5	9.4%	15.6%
Indigenous	3	5.7%	9.4%
Other	21	39.6%	65.6%
Not Reported	21	39.6%	

Note. Population composition was assessed using studies that provided extractable data on MSM-specific and total sample sizes. Usable sample-size data were available for 52 out of 53 studies; one modeling study without a numeric sample size was excluded. Ethnicity was summarized at the study level because the extraction sheet recorded ethnicity categories by article rather than by individual participant counts. As a result, ethnicity categories reflect the number of studies reporting each category, not the combined ethnic composition of all participants. Ethnicity data were reported in 32 of the 53 studies. A single study could contribute to more than one ethnicity category, so the percentages do not add up to 100%. The denominator for the “% of studies reporting ethnicity” includes only those studies that reported ethnicity data. NR = not reported; MSM = men who have sex with men.

**Table 2:** Summary of study-level uptake–willingness clusters and implementation interpretation.

Cluster	No. of Studies	Study Locations	Study Designs	Population	Main Interpretation
High vaccination rate & low willingness	1	France (1)	Mixed-methods study (1)	MSM (1)	This pattern may indicate that existing service pathways, provider engagement, or policy support enabled uptake despite remaining gaps in willingness, trust, or perceived risk. It should be interpreted cautiously because it is based on one study.
Low vaccination rate & low willingness	4	United States (1); Ireland (1); Taiwan (1); Mainland China (1)	Cross-sectional studies (3); cohort study (1)	MSM (4)	This cluster reflects a combined demand-and-access challenge. Knowledge gaps, low perceived susceptibility, cost concerns, provider communication gaps, and restrictive eligibility or coverage policies may jointly constrain both vaccination intention and actual uptake.
Low vaccination rate & high willingness	3	Italy (1); France (2)	Cross-sectional studies (3)	MSM (2); Mixed sexual/gender minority sample (1)	This cluster most clearly suggests an implementation gap. Willingness did not translate into vaccination, likely because of access barriers, safety or effectiveness concerns, insufficient provider recommendation, service delivery constraints, and policy restrictions.
High vaccination rate & high willingness	1	England (1)	Cross-sectional study (1)	Mixed sexual/gender minority sample (1)	This pattern may represent a more enabling vaccination context, with stronger alignment between individual acceptance, provider or system support, and policy access. Because only one study was included, it should be treated as a descriptive signal rather than a stable cluster profile.

Note. This table summarizes the nine studies included in the exploratory uptake–willingness clustering analysis, defined as studies with extractable data on both HPV vaccination uptake and willingness. Cluster labels describe relative study-level patterns and should be interpreted descriptively rather than as population-level prevalence estimates or causal conclusions. Detailed study-level barriers and opportunity themes are provided in Supplementary Table S5. Where numbers appear in parentheses, they indicate the number of studies within each cluster that reported the corresponding theme, not participant-level prevalence. HPV = human papillomavirus; MSM = men who have sex with men; Mixed sexual/gender minority samples = study sample including MSM together with at least one other sexual and/or gender minority subgroup, such as GBM, transgender women, non-binary/gender-diverse participants, or broader LGBTQ+ participants; NA = not applicable/no corresponding data. Two included studies focused on biological or clinical HPV outcomes, including anal HPV genotype prevalence and HPV antibody/immunogenicity, and were therefore not included in the barrier or opportunity theme mapping (Supplementary Table S2 extraction IDs 282 and 285).

**Table 3:** Corresponding barrier and opportunity themes across SEM levels.

SEM Level	Corresponding Theme	Barrier	Opportunity
Individual Level	Knowledge & Risk Perception	Knowledge gaps	HPV knowledge/risk appraisal
	Vaccine Beliefs	Safety/Side effect concerns	Confidence in vaccine efficacy/trust
Interpersonal Level (Provider-related)	Provider Recommendation	Lack of recommendation	Proactive provider endorsement
	Clinical Relationship	Poor patient–provider communication	Trusting clinical relationship/disclosure
Organizational Level	Service Delivery	Service delivery constraints	Integrated and easy-to-access vaccination services
Community Level	Social Norms	Stigma & restrictive social norms	Social influence & community support
	Information Environment	Weak awareness environment	Strong media & information environment
Policy/Societal Level	Policy Eligibility	Eligibility restrictions	Inclusive vaccination policies
	Affordability	High out-of-pocket costs	Financial accessibility
	Structural Access	Structural access inequities	Equitable vaccine distribution & coverage

Note. SEM = socio-ecological model.

## Data Availability

This study is a systematic review based on published data. All data extracted from the included studies, along with the statistical code used for the analyses, are available from the corresponding author (YWK) upon reasonable request.

## Supplementary Materials

The following supporting information can be downloaded at https://www.mdpi.com/article/10.3390/vaccines14070632/s1. Supplementary Table S1. Complete Database-Specific Search Strategies Used for the Systematic Review; Supplementary Table S2. Characteristics and extracted data of the included studies; Supplementary Table S3. Definitions and examples of socio-ecological model (SEM) subthemes; Supplementary Table S4. Definitions of Uptake and Willingness Used in the Exploratory Clustering Analysis; Supplementary Table S5. Detailed Study-Level Summary of Exploratory Uptake–Willingness Clusters and Associated Barrier and Opportunity Themes; Supplementary Figure S1. Methodological quality and risk-of-bias assessment by study design; Supplementary Figure S2. Item-level methodological quality assessment of included studies by study design.
