# Supplementary material for: Opportunities and Barriers to HPV Vaccination Among Men Who Have Sex with Men and Related Sexual and Gender Minority Populations: A Systematic Review and Exploratory Clustering Analysis Using a Socio-Ecological Framework
Source: Vaccines (Basel). 2026 Jul 20;14(7):632. doi: 10.3390/vaccines14070632 (PMC13431308; doi:10.3390/vaccines14070632)
Supplement: Supplementary file 1 [file vaccines-14-00632-s001.zip › Supplementary Figure S1.pptx]

## Slide 1
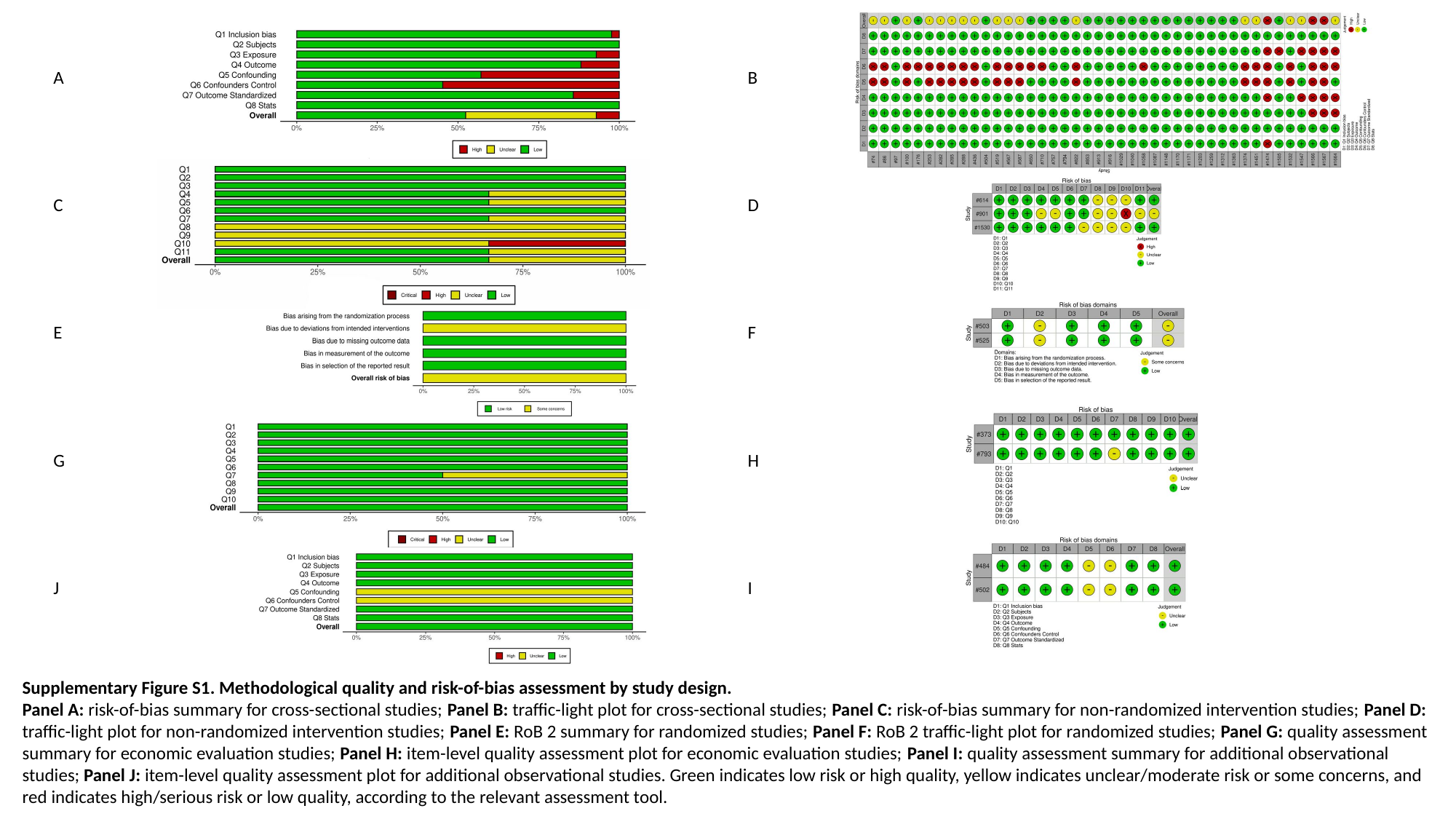

A
B
C
D
E
F
G
H
J
I
Supplementary Figure S1. Methodological quality and risk-of-bias assessment by study design.
Panel A: risk-of-bias summary for cross-sectional studies; Panel B: traffic-light plot for cross-sectional studies; Panel C: risk-of-bias summary for non-randomized intervention studies; Panel D: traffic-light plot for non-randomized intervention studies; Panel E: RoB 2 summary for randomized studies; Panel F: RoB 2 traffic-light plot for randomized studies; Panel G: quality assessment summary for economic evaluation studies; Panel H: item-level quality assessment plot for economic evaluation studies; Panel I: quality assessment summary for additional observational studies; Panel J: item-level quality assessment plot for additional observational studies. Green indicates low risk or high quality, yellow indicates unclear/moderate risk or some concerns, and red indicates high/serious risk or low quality, according to the relevant assessment tool.
