# Supplementary material for: Opportunities and Barriers to HPV Vaccination Among Men Who Have Sex with Men and Related Sexual and Gender Minority Populations: A Systematic Review and Exploratory Clustering Analysis Using a Socio-Ecological Framework
Source: Vaccines (Basel). 2026 Jul 20;14(7):632. doi: 10.3390/vaccines14070632 (PMC13431308; doi:10.3390/vaccines14070632)
Supplement: Supplementary file 1 [file vaccines-14-00632-s001.zip › Supplementary Figure S2.pptx]

## Slide 1
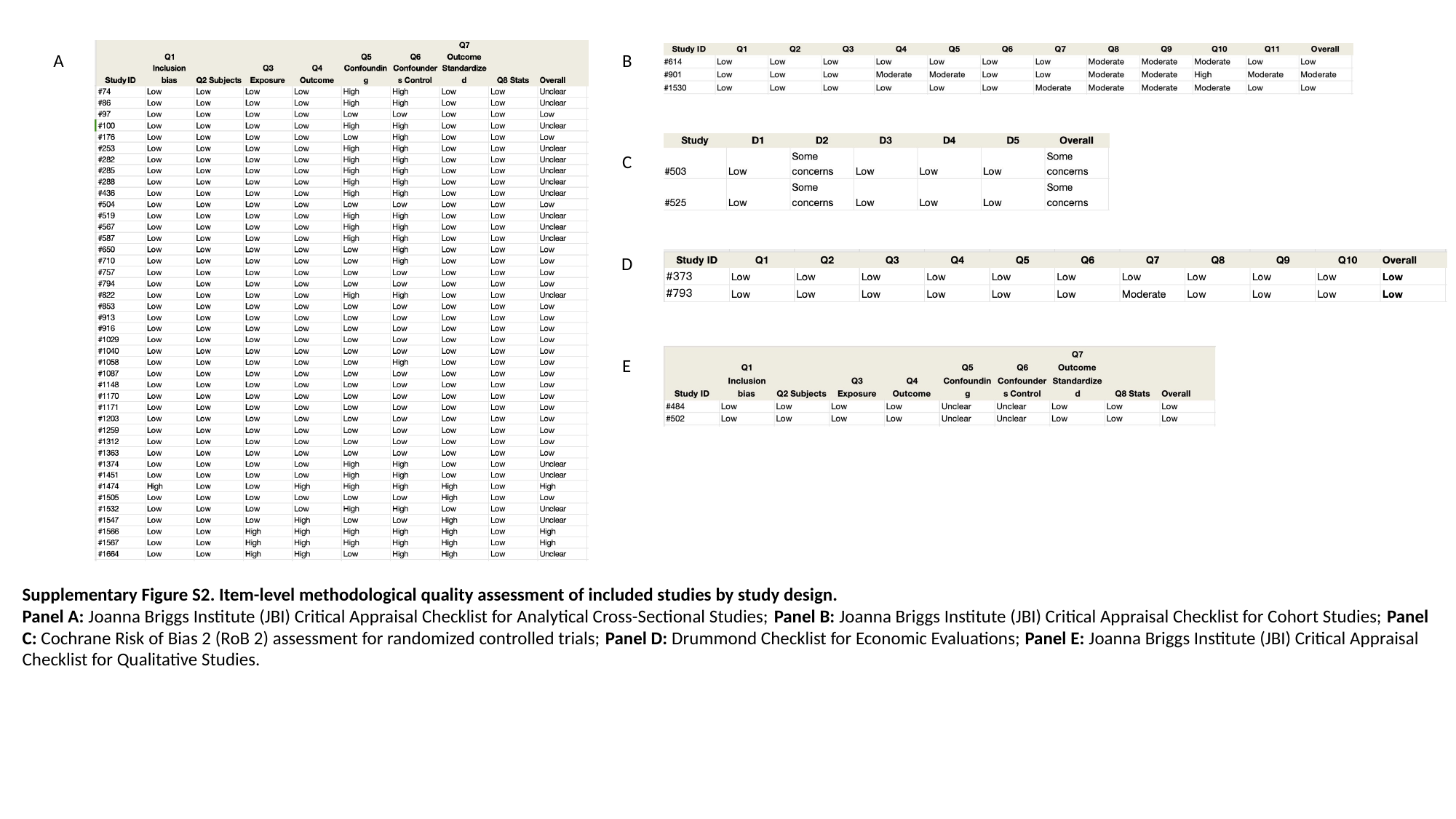

A
B
C
D
E
Supplementary Figure S2. Item-level methodological quality assessment of included studies by study design.
Panel A: Joanna Briggs Institute (JBI) Critical Appraisal Checklist for Analytical Cross-Sectional Studies; Panel B: Joanna Briggs Institute (JBI) Critical Appraisal Checklist for Cohort Studies; Panel C: Cochrane Risk of Bias 2 (RoB 2) assessment for randomized controlled trials; Panel D: Drummond Checklist for Economic Evaluations; Panel E: Joanna Briggs Institute (JBI) Critical Appraisal Checklist for Qualitative Studies.
