# Supplementary material for: Opportunities and Barriers to HPV Vaccination Among Men Who Have Sex with Men and Related Sexual and Gender Minority Populations: A Systematic Review and Exploratory Clustering Analysis Using a Socio-Ecological Framework
Source: Vaccines (Basel). 2026 Jul 20;14(7):632. doi: 10.3390/vaccines14070632 (PMC13431308; doi:10.3390/vaccines14070632)
Supplement: Supplementary file 1 [file vaccines-14-00632-s001.zip › Supplementary Table S1.pdf]

Supplementary Table S1. Complete Database-Specific Search Strategies Used for the Systematic Review.

Database / platform	Search strategy	Limits and filters	Search date	Records retrieved
**PubMed**	("papillomavirus vaccines"[MeSH Terms] OR "Papillomavirus Vaccine"[Title/Abstract] OR "Human Papillomavirus Vaccines"[Title/Abstract] OR "Human Papillomavirus Vaccine"[Title/Abstract] OR "HPV Vaccine"[Title/Abstract] OR "HPV Vaccines"[Title/Abstract]) AND ("male"[Title/Abstract] OR "men"[Title/Abstract])	Publication date: 1 January 2010 to 31 December 2025. Language filter: English or Chinese. Text availability: Full text.	24 January 2026	1,570
**Web of Science Core Collection**	AB=("papillomavirus vaccine*" OR "human papillomavirus vaccine*" OR "HPV vaccine*") AND TS=(male OR men)	Timespan: 2010–2025. Language filter: English. Document type: Article.	25 January 2026	1,282
**Scopus**	TITLE-ABS-KEY("papillomavirus vaccine*" OR "human papillomavirus vaccine*" OR "HPV vaccine*") AND TITLE-ABS-KEY(male OR men)	Publication year: 2010–2025. Language filter: English or Chinese. Document type was not used as a search restriction; study design eligibility was applied during screening.	24 January 2026	339
**Ovid MEDLINE**	("papillomavirus vaccines" or papillomavirus vaccine* or human papillomavirus vaccine* or HPV vaccine*) AND (male or men)	Publication year: 2010–2025; language: English; Full-text restriction	25 January 2026	100
**CNKI**	篇关摘=(HPV 疫苗 OR 人乳头瘤病毒疫苗 OR 人类乳头瘤病毒疫苗) AND 篇关摘=(男性 OR 男性人群)	Publication date: 1 January 2010 to 31 December 2025. Field searched: 篇关摘 (title/keywords/abstract). Matching mode: exact. Chinese–English term expansion	25 January 2026	184

		enabled; synonym expansion not selected. Language eligibility: Chinese-language records.		
**WanFang Data**	主题=(HPV 疫苗 OR 人乳头瘤病毒疫苗 OR 人类乳头瘤病毒疫苗) AND 主题=(男性 OR 男性人群)	Publication date: 1 January 2010 to 31 December 2025. Literature types: journal articles, dissertations/theses. Language eligibility: Chinese.	25 January 2026	50

Note. *Search counts indicate the number of records retrieved and exported from each database on the original search date before deduplication. Since bibliographic databases are continuously updated and search interfaces may change, exact counts may differ if the searches are rerun later. English and Chinese were not used as Boolean search terms. Language eligibility was applied using database language filters where available and otherwise during screening and full-text assessment.*
