# Supplementary material for: Opportunities and Barriers to HPV Vaccination Among Men Who Have Sex with Men and Related Sexual and Gender Minority Populations: A Systematic Review and Exploratory Clustering Analysis Using a Socio-Ecological Framework
Source: Vaccines (Basel). 2026 Jul 20;14(7):632. doi: 10.3390/vaccines14070632 (PMC13431308; doi:10.3390/vaccines14070632)
Supplement: Supplementary file 1 [file vaccines-14-00632-s001.zip › Supplementary Table S3.pdf]

Supplementary Table S3. Definitions and examples of socio-ecological model (SEM) subthemes

No.	SEM domain	Type	Subtheme	Working definition
1	Individual level	Opportunity	HPV knowledge & risk appraisal	HPV-related knowledge and risk perception, such as understanding transmission and infection, perceiving personal susceptibility, and recognizing severity (e.g., cancer, genital warts), etc
2			Positive vaccine beliefs & trust	Belief in vaccine efficacy and general pro-vaccine attitudes, such as trust in cancer/warts prevention, willingness to accept the vaccine, and positive views on safety, etc
3			Prevention motivation & vaccine acceptance	Motivation to prevent infection and protect others, such as the desire to protect oneself and sexual partners, and the intention to prevent future HPV infection, etc
4			Behavioral & socioeconomic enablers	Behavioral and socioeconomic factors facilitating vaccination, such as high-risk sexual behaviors, engagement in HIV prevention (e.g., PrEP), higher education, and income, etc
5		Barrier	Safety & side effects	Safety and side effect concerns, such as: fear of side effects, fainting from the injection, and uncertainty about long-term impacts, etc
6			Knowledge gaps & misconceptions	Knowledge gaps and misunderstandings, such as: believing that it is only targeted at women, not knowing that one meets the requirements, and insufficient understanding of the transmission channels, etc
7			High cost & economic barriers	High costs and economic obstacles, such as: vaccines being too expensive, low income, unemployment and other financial problems
8			Low perceived risk & susceptibility	Low perceived risk, such as: believing oneself to be at no risk, feeling old or already infected and not needing vaccination
9			Sociodemographic & behavioral factors	Sociodemographic and behavioral factors, such as specific age groups, educational attainment, rural residence, high-risk sexual behavior or multiple sexual partners and other associated factors
10			Effectiveness & trust issues	Efficacy and trust issues, such as: doubts about vaccine efficacy, interference from different medical opinions, etc
11			Stigma & disclosure	Stigmatization and privacy concerns, such as: fear of exposing sexual orientation, fear of being regarded as promiscuous, and embarrassment when discussing sexual health, etc
12			Logistical & time constraints	Logistical and time constraints, such as: no time, finding multiple vaccinations troublesome, rarely visiting the clinic, etc
13	Provider-related interpersonal level	Opportunity	Provider recommendation & active endorsement	Active recommendation and endorsement by healthcare providers, such as initiating HPV vaccine discussions and strongly endorsing vaccination, etc
14			Positive patient-provider communication	Open and effective communication regarding vaccination, such as opportunities to ask questions, shared

No.	SEM domain	Type	Subtheme	Working definition
				decision-making, and discussing benefits/eligibility, etc
15			Trusting clinical relationships	Trust and comfort in clinical interactions, such as disclosing MSM identity, comfort discussing sexual behavior, and LGBTQ+-Affirming interactions, etc
16			Provider competence & engagement	Provider knowledge and proactive involvement, such as awareness of MSM-specific risks and proactive promotion of the vaccine, etc
17		Barrier	Lack of provider recommendation or offer	Lack of recommendation or provision by medical providers: such as doctors not actively recommending, missing opportunities, not providing vaccines, etc
18			Poor patient-provider communication & non-disclosure	Poor doctor-patient communication and non-disclosure: such as doctors not knowing the patient's sexual orientation, lack of sexual health discussions, patients concealing their identities, etc.
19			Insufficient provider knowledge & engagement	Insufficient knowledge and low participation of medical providers: such as lack of regular HPV counseling, insufficient education for doctors, reliance on doctors to acquire knowledge but doctors not being proactive enough, etc
20			Stigma & Lack of LGBTQ+-Affirming Care	Stigmatization, discrimination and lack of LGBT literacy: such as homophobic doctors, lack of relevant training, patients' concerns about privacy leakage or being judged, etc
21			Healthcare access & logistical barriers	Medical resource acquisition and logistical obstacles: such as difficulty in making appointments, lack of knowledge of vaccination locations, absence of dedicated MSM free/subsidized programs, and having to go to private clinics, etc
22	Organizational/institutional level	Opportunity	Integrated service delivery	Integration of vaccination into existing services, such as combining with HIV/STI services (e.g., VCT clinics), etc
23			Accessible & convenient vaccination services	Availability of convenient services, such as school-based programs or accessible clinic hours, etc
24			Proactive vaccination infrastructure	System-level support for vaccination, such as national/local campaigns and organized delivery systems, etc
25		Barrier	Workforce Training & Capacity	Institutional knowledge, staff training, and organizational capacity to support HPV vaccination delivery, including workforce education, clinical guidance, and continuing professional development.
26			Clinical Workflow & System Design	Organizational processes and system design that influence HPV vaccination implementation, such as clinical workflows, referral pathways, appointment scheduling, documentation systems, care coordination, and integration of vaccination into routine services.
27			Service Delivery Constraints	Operational barriers that limit the delivery of HPV vaccination services, such as staff shortages, limited consultation time, inadequate

No.	SEM domain	Type	Subtheme	Working definition
				resources, vaccine supply issues, workload, and service availability.
28	Community level	Opportunity	Information & media environment	Exposure to health information via media, such as social media, internet-based info, and public awareness campaigns, etc
29			Cues to action & emotional triggers	Triggers prompting vaccination behavior, such as HPV-related worry (cancer/warts) and external prompts from peers or media, etc
30			Social influence & community support	Support and influence from social networks, such as peer recommendations, family influence, and MSM community attachment, etc
31			Information & education channels	Channels for disseminating knowledge, such as community-level information flow and peer discussions, etc
32		Barrier	Structural prevention context	Inequities in prevention benefits, such as herd protection inequity caused by female-only vaccination programs, etc
33			Stigma & social norms	Social stigma and conservative norms, such as MSM stigma, cultural/religious conservatism, and confidentiality concerns, etc
34			Knowledge & awareness environment	Lack of general knowledge in the community, such as lack of comprehensive sex education and low HPV awareness, etc
35			Social Network & Information Flow	Community information environments that shape HPV vaccination decisions, such as peer communication, partner discussions, community organization outreach, social media exposure, and information sharing within MSM networks.
36	Policy/societal level	Opportunity	Inclusive vaccination policy	Policies supporting equal access, such as gender-neutral programs, inclusion of MSM in strategies, and age expansion, etc
37			Targeted & catch-up programs	Specific programs for target groups, such as MSM-specific initiatives and time-limited catch-up vaccination, etc
38			Financial accessibility	Financial support mechanisms, such as free programs, subsidies, insurance coverage, and reduced prices, etc
39			Public health delivery infrastructure	Infrastructure supporting delivery, such as school-based programs and integration with sexual health services, etc
40			Policy, access & cost opportunities	Broad opportunities related to policy and cost, such as removing financial barriers and ensuring inclusive access, etc
41		Barrier	Eligibility & policy restrictions	Policy-based limitations, such as female-only programs, age limits, lack of gender-neutral policies, and risk-based eligibility, etc
42			Financial & coverage barriers	Economic obstacles, such as high out-of-pocket costs, limited insurance coverage, and lack of public funding, etc
43			Structural access inequities	Unequal access to resources, such as geographic variation and unequal resource allocation, etc
