# Supplementary material for: Opportunities and Barriers to HPV Vaccination Among Men Who Have Sex with Men and Related Sexual and Gender Minority Populations: A Systematic Review and Exploratory Clustering Analysis Using a Socio-Ecological Framework
Source: Vaccines (Basel). 2026 Jul 20;14(7):632. doi: 10.3390/vaccines14070632 (PMC13431308; doi:10.3390/vaccines14070632)
Supplement: Supplementary file 1 [file vaccines-14-00632-s001.zip › Supplementary Table S4.pdf]

Supplementary Table S4. Definitions of Uptake and Willingness Used in the Exploratory Clustering Analysis

Article No.	Author / Study	Cluster	Uptake definition used in this review	Operational definition of willingness used in this review
#79	Annequin et al.	**High vaccination rate & Low willingness**	The proportion of participants who had received at least one dose of the HPV vaccine.	Near-term vaccination intention among unvaccinated MSM using PrEP. The positive indicator was intention to receive HPV vaccination "as soon as possible"; where vaccine cost was addressed, this included willingness even without reimbursement. This represents near-term intention in a PrEP-using MSM subgroup rather than unconditional willingness in all MSM.
#86	Armstrong et al.	**High vaccination rate & High willingness**		Provider-recommendation-conditioned willingness among participants who were unvaccinated, incompletely vaccinated, or unsure of their HPV vaccine dose history. The positive indicator was agreement with the statement that they would receive the HPV vaccine if recommended by a doctor or healthcare provider. This represents conditional willingness rather than unconditional vaccination intention.
#176	Brosset et al.	**Low vaccination rate & High willingness**		The original study did not directly report HPV vaccination willingness or acceptability. For clustering, the available HPV-specific vaccine hesitancy measure was used as a conservative acceptability proxy. This proxy was based on HPV-specific hesitancy items, including perceived HPV disease severity, fear of HPV infection, perceived HPV vaccine effectiveness, and concern about severe vaccine side effects. It should be interpreted as a proxy indicator rather than a directly measured willingness rate.
#519	Giuliani et al.	**Low vaccination rate & High willingness**		Willingness among MSM who had heard of the HPV vaccine. The positive indicator was answering "Yes" to willingness to be vaccinated. Because the denominator was limited to HPV-vaccine-aware MSM, this represents awareness-conditioned willingness rather than willingness in the full MSM sample.
#822	Lueck et al.	**Low vaccination rate & Low willingness**		Composite vaccine acceptance. Participants were classified as accepting HPV vaccination if they had already received the HPV vaccine or strongly agreed that they planned to receive the HPV vaccine in the next six months. Because this measure includes both past vaccination behavior and future intention, it was treated as a composite acceptance indicator rather than a pure prospective willingness measure.
#1087	Petit & Epaulard	**Low vaccination rate & High willingness**		Acceptability among participants who reported being unvaccinated against HPV. The positive indicator was answering "rather yes" or "definitely yes" when asked whether they would accept HPV vaccination. This represents acceptability among unvaccinated participants.
#1203	Sadlier et al.	**Low vaccination rate & Low willingness**		Unconditional near-term vaccination intention. The positive indicator was answering "Yes" to the question asking whether participants planned to take up HPV vaccination in the next six months. Conditional acceptability under stated efficacy and cost scenarios was reported in the original study but was not used as the main clustering input.
#1530	Yao et al.	**Low vaccination rate & Low willingness**		Price-resistant near-term vaccination intention. The positive indicator was intention to receive HPV vaccination within six months regardless of price, corresponding to willingness even under the highest reported price condition. This represents willingness under a high-cost scenario and is therefore more conservative than free-vaccine acceptability.
#1532	Ye et al.	**Low vaccination rate & Low willingness**		Free-vaccine acceptability. The positive indicator was willingness to receive the HPV vaccine if it were available free of charge. This represents acceptability under a no-cost condition rather than unconditional willingness under real-world cost conditions.

Note. *This table summarizes the operational indicators used for the nine studies included in the exploratory uptake–willingness clustering analysis. Uptake was harmonized by the review team as receipt of at least one dose of HPV vaccine. Where original studies reported broader vaccination status, partial vaccination, completion status, or self-reported receipt of HPV vaccination, the clustering input was harmonized to this ≥1-dose definition when extractable. In contrast, willingness, acceptability, and intention were not measured uniformly across the primary studies. The willingness/acceptability inputs therefore represent the closest available study-level indicators in each article, including unconditional vaccination intention, near-term intention, willingness under free or high-cost vaccine conditions, provider-recommendation-conditioned willingness, composite vaccine acceptance, and a vaccine-hesitancy-based proxy indicator. Differences in denominators and conditions, such as unvaccinated participants only, HPV-vaccine-aware participants only, provider recommendation, or vaccine cost, are described in the table. These values were used only to support an exploratory study-level pattern analysis and should not be interpreted as fully standardized willingness prevalence estimates, participant-level prevalence, or causal effects. HPV = human papillomavirus; MSM = men who have sex with men; MSM-PrEP = men who have sex with men using pre-exposure prophylaxis.*
