# Supplementary material for: Opportunities and Barriers to HPV Vaccination Among Men Who Have Sex with Men and Related Sexual and Gender Minority Populations: A Systematic Review and Exploratory Clustering Analysis Using a Socio-Ecological Framework
Source: Vaccines (Basel). 2026 Jul 20;14(7):632. doi: 10.3390/vaccines14070632 (PMC13431308; doi:10.3390/vaccines14070632)
Supplement: Supplementary file 1 [file vaccines-14-00632-s001.zip › Supplementary Table S5.pdf]

Supplementary Table S5. Detailed Study-Level Summary of Exploratory Uptake–Willingness Clusters and Associated Barrier and Opportunity Themes.

Cluster	No. of studies	Total sample size	Study Locations	Study design	Population core	Top individual barriers	Top provider/interpersonal barriers	Top organizational barriers	Top community barriers	Top policy barriers	Top opportunities
High vaccination rate & Low willingness	1	354	France	Mixed-methods study	MSM	Effectiveness & Trust Issues (1); Knowledge Gaps & Misconceptions (1); Low Perceived Risk & Susceptibility (1)	Lack of Provider Recommendation or Offer (1); Poor Patient-Provider Communication & Non-disclosure (1); Stigma (1)	Clinical Workflow & System Design (1)	NA	Financial & Coverage Barriers (1)	Provider Recommendation & Active Endorsement (1); Positive Patient–Provider Communication (1); Trusting Clinical Relationships (1); Inclusive Vaccination Policy (1)
Low vaccination rate & Low willingness	4	838	United States; Ireland; Taiwan; Mainland China	Cross-sectional study (3); cohort study	MSM (4)	Knowledge Gaps & Misconceptions (2); Low Perceived Risk & Susceptibilit	Lack of Provider Recommendation or Offer (1); Poor Patient-Provider Communication & Non-	Clinical Workflow & System Design (1)	Structural Prevention Context (2); Stigma & Social Norms (1)	Financial & Coverage Barriers (4); Eligibility & Policy Restrictions (4);	Positive Vaccine Beliefs & Trust (4); HPV Knowledge & Risk Appraisal (3); Prevention Motivation & Vaccine Acceptance (3);

						y (2); High Cost & Economic Barriers (2)	disclosure (1); Stigma (1)			Structural Access Inequities (2)	Financial Accessibility (3)
Low vaccination rate & High willingness	3	6120	Italy; France (2)	Cross-sectional study (3)	Mixed sexual/gender minority sample; MSM (2)	Safety & Side Effects (3); Effectiveness & Trust Issues (2); Low Perceived Risk & Susceptibility (1)	Lack of Provider Recommendation or Offer (2); Poor Patient-Provider Communication & Non-disclosure (2)	Service Delivery Constraints (1)	Structural Prevention Context (1)	Eligibility & Policy Restrictions (3); Structural Access Inequities (1); Financial & Coverage Barriers (1)	HPV Knowledge & Risk Appraisal (3); Prevention Motivation & Vaccine Acceptance (1); Accessible & Convenient Vaccination Services (1); Integrated Service Delivery (1)
High vaccination rate & High willingness	1	246	England	Cross-sectional study	Mixed sexual/gender minority sample	Knowledge Gaps & Misconceptions (1); Logistical & Time Constraints (1); Safety & Side Effects (1)	Insufficient Provider Knowledge & Engagement (1); Lack of Provider Recommendation or Offer (1)	Stigma & Social Norms (1); Knowledge & Awareness Environment (1); Social Network &	NA	Eligibility & Policy Restrictions (1)	HPV Knowledge & Risk Appraisal (1); Positive Vaccine Beliefs & Trust (1); Provider Recommendation & Active Endorsement (1); Positive Patient–Provider Communication (1)

								Information Flow (1)			

*Note. This table summarizes the nine studies included in the uptake–willingness clustering analysis, defined as studies with extractable data on both HPV vaccination rate and willingness to receive HPV vaccination. Barrier and opportunity themes were coded at the study level; therefore, the numbers in parentheses indicate the number of studies within each cluster that reported the corresponding theme, not participant-level prevalence. For example, "Knowledge Gaps & Misconceptions (2)" indicates that this theme was reported in two studies within that cluster. A single study could contribute more than one theme within the same category. Two included studies focused on biological or clinical HPV outcomes, including anal HPV genotype prevalence and HPV antibody/immunogenicity, and were therefore not included in the barrier or opportunity theme mapping **(Supplementary Table S1** extraction IDs 282 and 285). NA indicates that no corresponding theme was extracted for that cluster/category. HPV = human papillomavirus; MSM = men who have sex with men; Mixed sexual/gender minority sample = study sample included MSM together with at least one other sexual and/or gender minority subgroup, such as GBM, transgender women, non-binary/gender-diverse participants, or broader LGBTQ+ participants. NA = not applicable/no corresponding data.*
